# Actions required to implement integrated care for older people in the community using the World Health Organization's ICOPE approach: A global Delphi consensus study

**DOI:** 10.1371/journal.pone.0205533

**Published:** 2018-10-11

**Authors:** Andrew M. Briggs, Islene Araujo de Carvalho

**Affiliations:** 1 Department of Ageing and Life Course, World Health Organization, Geneva, Switzerland; 2 Faculty of Health Sciences, Curtin University, Perth, Australia; University of Antwerp, BELGIUM

## Abstract

**Background:**

Integrated care is recognised as an important enabler to healthy ageing, yet few countries have managed to sustainably deliver integrated care for older people. We aimed to gather global consensus on the key actions required to realign health and long-term systems and integrate services to implement the World Health Organization (WHO) Integrated Care for Older People (ICOPE) approach.

**Methods:**

A two-round eDelphi study, including a global consultation meeting, was undertaken to identify, refine and generate consensus on the actions required across high-, middle- and low-income countries to implement the WHO ICOPE approach. In round 1, a framework of 31 actions, empirically derived from previous WHO evidence reviews was presented to panellists to judge the relative importance of each action (numeric rating scale; range:1–9) and provide free-text comments concerning the scope of the actions. These outcomes were discussed and debated at the global consultation meeting. In round 2, a revised framework of 19 actions was presented to panellists to measure their extent of agreement and identify ‘essential’ actions (five-point Likert scale; range: strongly agree to strongly disagree). A threshold of ≥80% for agree/strongly agree was set *a priori* for consensus.

**Results:**

After round 1 (n = 80 panellists), median scores across 31 actions ranged from 6 to 9. Based on pre-defined category thresholds for median scores, panellists considered 28 actions (90·3%) as ‘important’ and three (9·7%) as ‘uncertain’. Fifteen additional actions were suggested for inclusion based on free-text comments, creating 46 for consideration at the global consultation meeting. In round 2 (n = 84 panellists), agreement (agree or strongly agree) ranged from 84·6–97·6%, suggesting consensus. Fourteen (73·7%) actions were rated as essential.

**Conclusion:**

Fourteen essential actions and five important actions are necessary at system (macro; n = 10) and service (meso; n = 9) levels to implement community-based integrated care for older people.

## Introduction

The global population is ageing more rapidly than ever before–from 2015 to 2050, the proportion of the global population aged 60 years and over will nearly double [1]. This change will happen across high-, middle- and low-income settings, highlighting the need for a realigning health systems to the needs of the older populations they now serve [1]. Population ageing is largely attributed to longer life expectancy, lower fertility rates and increasing prevalence of chronic health conditions, which commonly manifest as multi-morbidities in older people [2]. Many other major social changes are occurring alongside population ageing, for example urbanization and globalization have been accompanied by increased migration, in which younger generations migrate to areas of growth, while older people are left in poorer rural areas without family structures and social safety. Collectively, these issues create new and complex challenges for health and long-term care systems.

There is an urgent need to realign health systems and build sustainable long-term care systems to better respond to the health and social care needs of older populations [3, 4]. Providing person-centred and integrated care and guaranteeing access to it will require systems to be organized around older people’s needs and preferences, and will require services to be age-friendly and closely engaged with families and communities [1]. The World Health Organization (WHO) defines integrated care as “services that are managed and delivered so that people receive a continuum of health promotion, disease prevention, diagnosis, treatment, disease-management, rehabilitation and palliative care services, coordinated across the different levels and sites of care within and beyond the health sector, and according to their needs throughout the life course” [4]. Integration initiatives therefore need to involve multiple sites and levels, including: system-level (macro), service/organisational-level (meso) and clinical-level (micro) activities [5, 6]. In the context of care delivery for older people, WHO operationalized this concept of integrated care through the *Integrated Care for Older People* (ICOPE) approach [5]. The ICOPE approach is aimed at improving or maintaining older people’s intrinsic capacity and functional ability to support healthy ageing [1], through community- level health and social care interventions [3].

Integration of health and social care is widely advocated as a way to improve the management and outcomes for the increasing numbers of older people with varying and complex health needs [1, 7]. Despite this, the evidence for the effectiveness of integrated care approaches remains inconsistent across care settings [8–14] and there is variability in the elements within integrated care interventions [5, 15]. Support for implementation efforts of integrated care approaches such as ICOPE are needed to ensure sustainability and scalability beyond pilot initiatives and trials, and critically, that are transferable across economies and care settings. While evidence is accumulating to support implementation efforts [6, 16–18], there remains no global consensus concerning actions required at the service (meso) or system (macro) levels to achieve sustainable implementation of integrated care approaches for older people. The majority of research directed at enabling care integration has been undertaken at the micro (clinical) level [5, 19], with less attention devoted to supporting implementation at meso and macro levels. WHO has also undertaken research at the micro level through the development of clinical guidelines and is currently translating that evidence into practical support tools for community-based health workers [20]. Therefore, the aim of this study was to evaluate and generate consensus on specific actions required for implementation of the WHO ICOPE approach at the meso and macro levels.

## Methods

### Design

A two-round eDelphi study and global consultation meeting undertaken between Round 1 and Round 2, were used to address the research aim. Ethical approval to conduct the study was granted by the WHO Research Ethics Review Committee and participants provided consent to participate in the research. Specifically, participants enrolled in the study through an electronic survey platform (described below) where they could review a Participant Information Statement and consent or decline to participate by selecting an on-screen button.

### Sampling

A multinational and multidisciplinary panel of experts, service providers and policy makers in integrated care for older people was recruited by WHO in 2017. The panel was constructed to be representative across professional disciplines; diverse research, policy and service design and delivery foci; and across high-, middle- and low-income economies of the UN Member States. While we did not include individual older people themselves on the panel, since the intended primary users of the research outcomes were service providers, service managers and system managers; national and international organisations representing health and social care for older people were included.

#### Round 1

In constructing the Delphi panel for round 1, potential research, policymaker, practitioner and civil society panellists were identified from a number of sources, including the professional contact network of the WHO Department of Ageing and Life Course; the six WHO regional offices; and relevant academic journal publications on integrated care approaches for older people using a systematic search strategy published in aligned work [5]. Using these sampling methods, 150 individuals were identified to participate in Round 1. Each received a direct email invitation from WHO to participate and respond to screening questions to determine eligibility and provide consent to participate.

Potential panellists were considered eligible to join the panel if they: 1) were currently involved in policy development or implementation related to health or social care for older people; or 2) had five or more years of professional engagement or experience in implementation of integrated care approaches; or 3) had published three or more peer-reviewed journal papers on integrated care approaches.

#### Global consultation meeting

A three-day global consultation was undertaken in Berlin in October 2017 (http://www.who.int/ageing/health-systems/icope/icope-consultation/en/) [21]. Panellists who participated in Round 1 (n = 80) and representatives from Member States and organisations that represented older people or were involved in health or social care delivery for older people were invited to participate in the global consultation (n = 49).

#### Round 2

Whereas the focus of Round 1 was largely exploratory in nature and aimed to evaluate the relative importance and content validity of an empirically-derived initial framework of ICOPE elements with a more narrow expert Delphi panel, Round 2 aimed to sample experts more broadly in order to comprehensively evaluate the acceptability of a revised framework of ICOPE actions developed as an outcome of the global consultation meeting. For this reason, the Round 2 panel was expanded beyond Round 1 participants.

Panellists from Round 1 were invited to participate in Round 2. The Round 2 panel was expanded from Round 1 by sampling WHO staff from across the globe to ensure that all relevant WHO program areas had the opportunity to contribute. The systematic search strategy used in sampling researchers for Round 1 was repeated for the time period between the end of Round 1 and commencement of Round 2 to identify any researchers with recently published papers who could participate in Round 2. 194 direct email invitations to participate in Round 2 were issued, consisting of 80 Round 1 participants, 60 WHO staff, 49 participants from the global consultation meeting who did not participate in Round 1, and 5 additional (first-author) research experts identified through an updated systematic literature search.

### eDelphi protocol and analysis

For each round, an online survey platform was created in LimeSurvey^TM^ software (LimeSurvey GmbH, Hamburg, Germany). The survey collected demographic information to characterise the panel at each phase and presented a framework of ICOPE elements.

In Round 1, a framework of 31 elements relating to implementation of the ICOPE approach in health and long-term care systems was presented to panellists. An ‘element’ was defined as ‘a component of care or an action that is implementable to enable integrated care for older people’. Elements were empirically-derived from existing WHO evidence reviews [1, 5, 7], a pre-established taxonomy of 59 elements of integrated care [15], the *WHO Framework on Integrated People Centred Health Services* [4], and through internal consultation within WHO. For each of the 31 elements presented, panellists were asked to rate its level of importance in implementation of the WHO ICOPE approach using a 9-point numeric rating scale (1 = lowest importance to 9 = highest importance). Panellists were also invited to suggest revisions to elements or additional elements in free-text fields.

The criteria of the RAND UCLA appropriateness method were used to analyse data from Round 1.[22] For each element, the panel median was categorized as follows: 1–3 as not important, 4–6 as equivocal and 7–9 as important. The threshold for panel agreement was determined *a priori* as ≥ 70% of panellists’ ratings within the same 3-point region (that is, 1–3, 4–6, or 7–9) as the observed median. An element was defined as “important” where the overall panel median score was ≥ 7 with level of agreement of ≥ 70% within the 3-point region 7–9. An element with a panel median of 4–6 or median with a consensus of ≤ 70% within the same 3-point region was defined as “uncertain” and flagged for discussion at the global consultation meeting. An element with a panel median of 1–3 and a level of agreement of ≥ 70% within the 3-point region 1–3, was defined as “unimportant” and was removed from the framework of elements. Free text comments were content-analysed using a standard summative content analysis approach [23]. The analysis was restricted to comments relating to specific elements within the framework of 31 presented, and/or comments relating to new elements for consideration. Each comment was reviewed and like comments combined to create a list of additional elements suggested by the panellists. All comments were considered valid and presented for consideration at the global consultation meeting.

In Round 2, panellists were asked to review a revised framework of ‘actions’, presented as a detailed consultation document (available on request from the authors), and rate their level of agreement with each action as a required action to implement the WHO ICOPE approach using a 5-point Likert scale (1 = strongly disagree to 5 = strongly agree). In round 1, the components of the framework were referred to as ‘elements’, whereas in round 2, they were re-framed as ‘actions’. Typically, 9 or 10-point numeric ratings scales are used to measure a continuous construct such as importance, whereas a 5-point Likert scale is more appropriate when measuring latent constructs such as agreement [24]. Where panellists responded with a rating of ≤3 for an action, they were prompted to provide an explanatory response in a free-text field. Where panellists provided a response of ≥4 for an action, they were asked to consider whether the action was essential (i.e. a fundamental requirement) or non-essential (i.e. important, but not essential) for implementing the ICOPE approach. Panellists were also asked to provide their level of agreement with the revised overall framework of actions using the same 5-point scale, optionally provide general free-text comments on the revised overall framework, and rate the revised framework according to the five dimensions of the Honeycomb Model of User Experiences, using the same 5-point Likert scale [25, 26]. Nominal responses were analyzed using frequency statistics. A pooled panel threshold of ≥80% for “agree [4]” or “strongly agree [5]” responses was set *a priori* for retaining actions or identifying if an action was ‘essential’. The free-text comments from round 2 were similarly content-analysed using a summative content-analysis approach [23]. Each response was reviewed and categorized by one author (AMB) as either ‘relating to the action’, ‘relating to implementation of the action’, ‘an overarching guiding principle’, or ‘other’. Data categorised as ‘relating to the action’ were used to refine the actions and these were verified by a second author (IAC). Data relating to any other category were used by WHO to develop an implementation toolkit. The design and thresholds for Round 2 was consistent with a previously used method to develop a system-level framework [26].

Descriptive statistics were used to characterize the panel at each stage. The authors and broader group of collaborators (see Acknowledgments) reviewed the framework at each round for clarity and meaningfulness.

### Global consultation

As part of the global consultation small group (n = 9–10), facilitated discussions were held to consider the quantitative and qualitative outcomes of Round 1. Each group was led by two WHO facilitators to consider clarity of the elements, the appropriateness of new elements suggested by panellists in Round 1 and interpret reasons for lack of consensus for some elements in Round 1.

## Results

**Fig 1:**
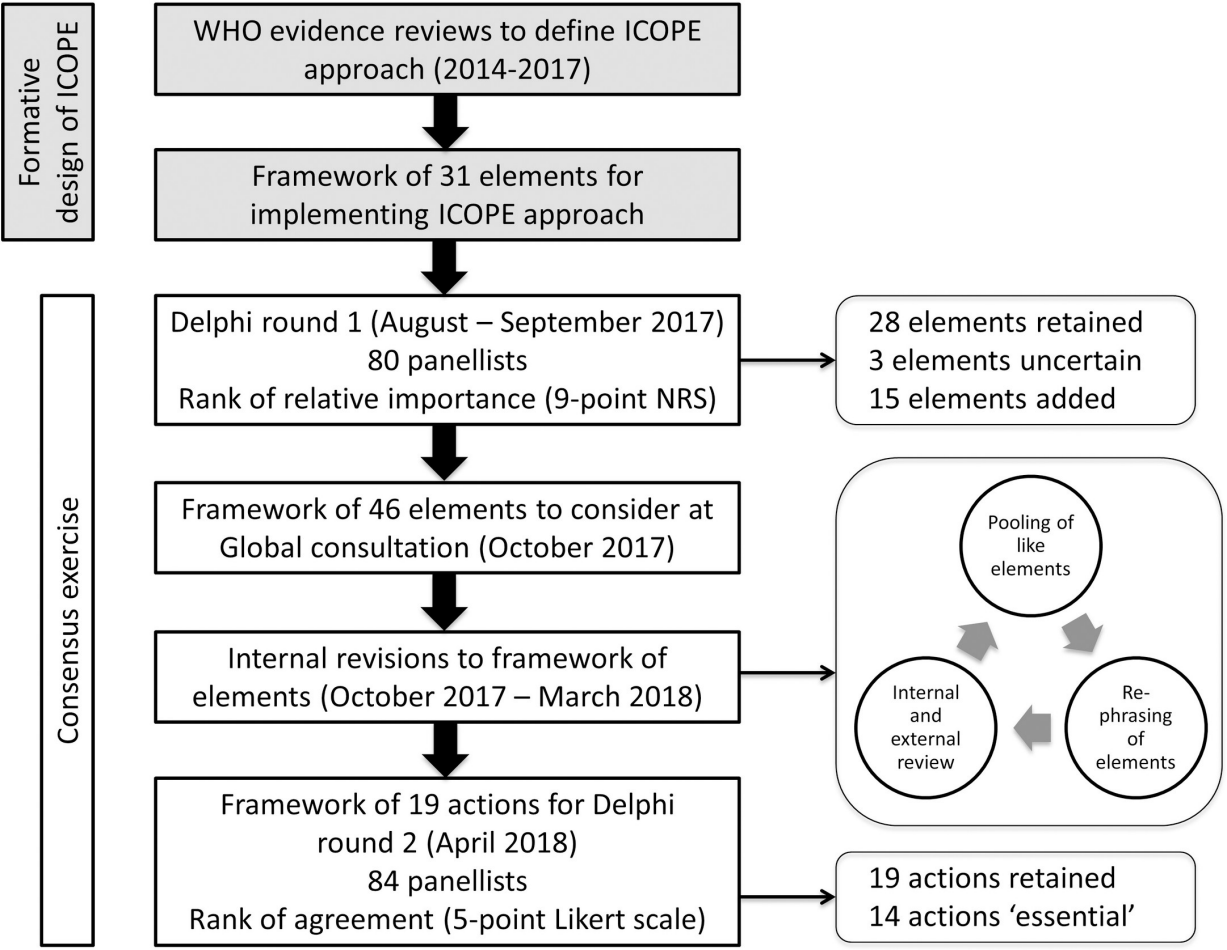
Chronological flowchart of the Delphi process, including stages of formative design of the ICOPE approach (grey shaded blocks).

Fig 1 summarises the stages and outcomes of the consensus exercise.

### Panel and global consultation characteristics

The demographic characteristics of the Delphi panels are summarised in Table 1. Of the 150 stakeholders invited to participate in Round 1, 85 (56·7%) participated, of which 80 (94%) were deemed eligible. In Round 2, 92 (47·4%) of the 194 invitees participated, of which 84 (91·3%) were deemed eligible. Of these, 35 (41·7%) and 40 (47·6%) also participated in Round 1 and global consultation meeting, respectively.

**Table 1:** Descriptive characteristics of the Delphi panels.

Descriptor	Round 1 panel	Round 2 panel
N (% female)	80 (47·5)	84 (42·9)
N (%) retained from round 1	-	35 (41·7)
Age–mean (SD) years [range]	49·4 (10·3) [27–70]	52·5 (10·3) [30–76]
Profession experience in integrated care–mean (SD) years [range]	15·7 (9·3) [1–35]	16·3 (10·4) [0–42]
Primary professional group: n (%)		
Researchers/academic	31 (38·8)	33 (39·3)
Program manager or implementer	19 (23·8)	8 (9·5)
Policy makers	13 (16·2)	8 (9·5)
Healthcare professionals	13 (16·2)	21 (25·0)
Non-government organization officer	0 (0)	7 (8·3)
Other	4 (5·0)	7 (8·3)
Geographic income band^: n (%)		
Low-income	8 (10·0)	5 (6·0)
Middle-income	16 (20·0)	30 (35·7)
High-income	56 (70·0)	49 (58·3)
Geographic region*: n (%)		
Africa	15 (18·7)	8 (9·5)
Asia	17 (21·2)	25 (29·8)
Central America	0 (0)	3 (3·6)
Eastern Europe	0 (0)	0 (0)
European Union	27 (33·8)	28 (33·3)
Middle East	2 (2·5)	2 (2·4)
North America	11 (13·8)	10 (11·9)
Oceania	7 (8·8)	6 (7·1)
South America	1 (1·2)	1 (1·2)
The Caribbean	0 (0)	1 (1·2)

^ based on World Bank country classifications by income level: 2017–2018 (https://datahelpdesk.worldbank.org/knowledgebase/articles/906519-world-bank-country-and-lending-groups)

* based on UN country grouping (https://www.internetworldstats.com/list1.htm#geo)

81 delegates participated in the global consultation meeting, of which 31 (38·3%) participated in Round 1. Delegates represented international research or service delivery experts (n = 37, 45·7%), Member States (n = 28, 34·6%) and WHO technical and support staff (n = 16, 19·7%).

### Round 1 outcomes

Across the 31 elements, median scores ranged from 6 to 9 and agreement within pre-specified median bands from 51·2–96·2%. Based on RAND UCLA criteria, panellists considered 28 of the 31 elements (90·3%) as important and three (9·7%) elements as uncertain after Round 1 and flagged for discussion at the global consultation meeting (#12: use of provider report cards; #18: integration of traditional and complementary medicines into health services; #21: development of new work cadres). No elements were ranked as unimportant (S1 Table). Compared to a pooled sample, panellists from high-income settings considered two additional elements (#2: active case finding; #19: establishment of performance management practices) as uncertain (n = 5 uncertain in total, 16.1%), whereas panellists from middle- and low-income settings considered only one element each as uncertain (n = 1, 3·2% and n = 1, 3·2%; respectively). In analysing free-text responses, an additional 15 elements were proposed (S1 File), culminating in 28 definitive elements, 3 uncertain elements and 15 potential additional elements for consideration at the end of Round 1 (N = 46). The additional 15 elements represented 7 themes, including: greater emphasis on ICT systems; specialised hospital and out of hospital services (public or private); outcomes-based funding models; workforce capacity building strategies; clinical guidelines to support care delivery; long-term care insurance; and age-friendly infrastructure.

### Global consultation outcomes

The 46 elements from Round 1 were considered and interpreted at the global consultation meeting. In discussing the three uncertain elements from round 1, participants concluded these elements were important considerations for ICOPE but needed to better contextualised in an overall approach to the implementation of ICOPE. Delegates recommended re-framing of all the elements as implementation-based action statements, pooling of like elements, and categorisation of elements into logical domains [21]. The WHO technical unit undertook a phased revision process, including: reducing redundancy across the 46 elements by pooling like-elements, grouping elements according to the *WHO Framework on integrated people-centred health services* [4], re-framing elements into action statements (henceforth, we refer to the components of the framework as ‘actions’, rather than ‘elements’) and undertaking an internal and external review of a revised framework of 19 actions.

### Round 2 outcomes

The pooled panel strength of agreement (agree or strongly agree) across the 19 actions ranged from 84·6–97·6%, suggesting consensus across the final set of all 19 (100%) actions; 9 (47·4%) targeted at the meso-level and 10 (52·6%) at the macro-level (Table 2). Similarly, all actions were scored as ≥80% agree and strongly agree by respondents in low-, middle- and high-income settings, other than action #18 (‘Utilise digital strategies to support self-management’) by high-income country respondents (79·5%; Table 2). Fourteen (73·7%) actions were rated as essential. Actions #10–12, #17 and #18 were ranked below the threshold for inclusion as essential by the pooled panel (range: 52·1–79·7%), largely due to responses from panellists in high-income settings.

**Table 2:** Delphi Round 2 outcomes for 19 actions. Actions are presented by domains of the *WHO Framework on integrated people centred health services (IPCHS)* and include an action title, target level for the health/social care system and a summative description. Responses presented as percentages (%) from 84 respondents, pooled and disaggregated by economic band.

No.	Action title [target level]	Action summary	Strongly disagree	Disagree	Uncertain	Agree	Strongly agree	Essential action
IPCHS Domain 1: Engaging and empowering people and communities								
1*	Services actively engage older people, their families and civil society in service delivery [meso]	Services need to implement processes to actively engage the community; i.e. older people, their families and civil society (e.g. non-government organisations) in the delivery of health and social care services to older people within the community.	P: 1·2	P: 0	P: 1·2	P: 14·3	P: 83·3	P: 100·0^#^
			LIC: 0	LIC: 0	LIC: 0	LIC: 0	LIC: 100·0	LIC: 100·0
			MIC: 3·3	MIC: 0	MIC: 0	MIC: 16·7	MIC: 80·0	MIC: 100·0
			HIC: 0	HIC: 0	HIC: 2·0	HIC: 14·3	HIC: 83·7	HIC: 100·0
2*	Services offer support and training for caregivers [meso]	Services should support the physical and mental wellbeing of caregivers and develop caregivers’ care competencies by offering combinations of training, support and respite care.	P: 2·4	P: 0	P: 0	P: 34·5	P: 63.1	P: 95·1^#^
			LIC: 0	LIC: 0	LIC: 0	LIC: 20·0	LIC: 80·0	LIC: 100·0
			MIC: 6·7	MIC: 0	MIC: 0	MIC: 26·7	MIC: 66·7	MIC: 100·0
			HIC: 0	HIC: 0	HIC: 0	HIC: 40·8	HIC: 59·2	HIC: 91·8
IPCHS Domain 2: Strengthening governance and accountability								
3*	Systems create or update policy and regulatory frameworks to support integrated care and protection for older people [macro]	Systems develop or update existing policy and regulatory frameworks to promote integrated health and social care and protection for older people. In particular, coordination of care between service delivery teams, care settings and levels of the health system are needed. Policy and regulatory frameworks should reflect the needs and priorities of local stakeholders. Policies/frameworks should be supported by a compelling ‘case for change’ to stimulate political will and leadership and support.	P: 1·2	P: 0	P: 6·0	P: 26·2	P: 66·7	P: 96.2^#^
			LIC: 0	LIC: 0	LIC: 0	LIC: 0	LIC: 100·0	LIC: 100·0
			MIC: 3·3	MIC: 0	MIC: 6·7	MIC: 36·7	MIC: 53·3	MIC: 92·6
			HIC: 0	HIC: 0	HIC: 6·1	HIC: 22·4	HIC: 71·4	HIC: 97·8
4*	Systems support active engagement of older people and their families, civil society and local service providers in policy and service development [macro]	Systems implement processes to actively engage and empower older people and their families, civil society (e.g. non-government organisations) and local service providers to participate in the development of health and social care policies, such as long-term care systems, and services for older people.	P: 1·2	P: 1·2	P: 1·2	P: 39·3	P: 57·1	P: 86·4^#^
			LIC: 0	LIC: 0	LIC: 0	LIC: 20·0	LIC: 80·0	LIC: 100·0
			MIC: 3·3	MIC: 0	MIC: 3·3	MIC: 33·3	MIC: 60·0	MIC: 85·7
			HIC: 0	HIC: 2·0	HIC: 0	HIC: 44·9	HIC: 53·1	HIC: 85·4
5*	Systems implement processes for quality assurance and improvement of health and social care services [macro]	Systems create and implement processes to measure quality of health and social care services and identify opportunities for quality improvement, aligned with evidence-based practice, and assessed with valid and reliable tools. For example, systems ideally collect information on patient/person-reported outcomes (PROMs), patient-reported experiences (PREMs), and service providers' performance and experiences.	P: 1·2	P: 0	P: 6·0	P: 36·9	P: 56·0	P: 87·2^#^
			LIC: 0	LIC: 0	LIC: 0	LIC: 0	LIC: 100·0	LIC: 80·0
			MIC: 3·3	MIC: 0	MIC: 0	MIC: 40·0	MIC: 56·7	MIC: 93·1
			HIC: 0	HIC:0	HIC: 10·2	HIC: 38·8	HIC: 51·0	HIC: 84·1
6*	Systems regularly review capacity to deliver care equitably and evaluate their performance [macro]	Regular capacity assessments and performance evaluations of the health and social care systems to deliver integrated care for all older people in a given setting are undertaken. Capacity assessments and performance evaluations should pay particular attention to disadvantaged groups with limited access.	P: 1·2	P: 0	P: 4·8	P: 46·4	P: 47·6	P: 87·3^#^
			LIC: 0	LIC: 0	LIC: 0	LIC: 40·0	LIC: 60·0	LIC: 80·0
			MIC: 3·3	MIC: 0	MIC: 0	MIC: 36·7	MIC: 60·0	MIC: 93·1
			HIC: 0	HIC: 0	HIC: 8·2	HIC: 53·1	HIC: 38·8	HIC: 84·4
IPCHS Domain 3: Reorienting the model of care								
7*	Services deliver care through a community-based workforce, supported by community-based services [meso]	Services deliver care through a community-based health and social care workforce, including paid and/or unpaid roles (e.g. family members), that is supported by complementary local services to deliver safe and effective care to older people in their home or community, where clinically appropriate and feasible.	P: 1·2	P: 0	P: 8·3	P: 26·2	P: 64·3	P: 94·7^#^
			LIC: 0	LIC: 0	LIC: 0	LIC: 0	LIC: 100·0	LIC: 100·0
			MIC: 3·3	MIC: 0	MIC: 6·7	MIC: 30·0	MIC: 60·0	MIC: 92·6
			HIC: 0	HIC: 0	HIC: 10·2	HIC: 26·5	HIC: 63·3	HIC: 95·5
8*	Services provide the necessary infrastructure to support safe and effective care delivery in the community [meso]	Services provide the necessary infrastructure (e.g. physical, transport, telecommunications) to enable safe and effective care delivery for older people in the community or their home. Wherever possible, existing infrastructure should be used.	P: 1·2	P: 0	P: 4·8	P: 34·5	P: 59·5	P: 94·9^#^
			LIC: 0	LIC: 0	LIC: 0	LIC: 40·0	LIC: 60·0	LIC: 100·0
			MIC: 3·3	MIC: 0	MIC: 3·3	MIC: 26·7	MIC: 66·7	MIC: 100·0
			HIC: 0	HIC: 0	HIC: 6·1	HIC: 38·8	HIC: 55·1	HIC: 91·3
9*	Provide services (and assistive products where required) that are acceptable to older people, effective and target functional ability^ [meso]	Services deliver safe and effective health and social care to improve functional ability^. Care is aligned to older people's needs, preferences, cultural practices, and supported by evidence. Assistive products, such as devices and rehabilitative and medical technologies are provided to improve functional ability^, as able.	P: 1·2	P: 1·2	P: 2·4	P: 35·7	P: 59·5	P: 88·8^#^
			LIC: 0	LIC: 0	LIC: 0	LIC: 0	LIC: 100·0	LIC: 100·0
			MIC: 3·3	MIC: 0	MIC: 6·7	MIC: 36·7	MIC: 53·3	MIC: 92·6
			HIC: 0	HIC: 2·0	HIC: 0	HIC: 38·8	HIC: 59·2	HIC: 85·4
10*	Systems utilise health information and communication technologies to facilitate information exchange between service providers [macro]	Where locally acceptable and feasible, systems implement health information and communication technologies and processes to facilitate storage, sharing and communication of information (e.g. health records, prescriptions, consultations) between health and social care providers and services. eHealth systems must be supported by appropriate data privacy and security policy and technology.	P: 1·2	P: 1·2	P: 3·6	P: 41·7	P: 52·4	P: 73·4
			LIC: 0	LIC: 0	LIC: 0	LIC: 40·0	LIC: 60·0	LIC: 80·0
			MIC: 3·3	MIC: 0	MIC: 0	MIC: 36·7	MIC: 60·0	MIC: 75·9
			HIC: 0	HIC: 2·0	HIC: 6·1	HIC: 44·9	HIC: 46·9	HIC: 71·1
11*	Systems collect and report data on intrinsic capacity** and functional ability^ of older adults within existing health information systems [macro]	Systems collect data on intrinsic capacity** and functional ability^ of older adults in health facilities and communities using existing health information systems as a means to improve quality and performance of health systems.	P: 1·2	P: 1·2	P: 3·6	P: 48·8	P: 45·2	P: 79·7
			LIC: 0	LIC: 0	LIC: 0	LIC: 0	LIC: 100·0	LIC: 100·0
			MIC: 3·3	MIC: 0	MIC: 0	MIC: 56·7	MIC: 40·0	MIC: 82·8
			HIC: 0	HIC: 2·0	HIC: 6·1	HIC: 49·0	HIC: 42·9	HIC: 75·6
IPCHS Domain 4: Co-ordinating services within and between sectors								
12*	Services actively seek to identify older people in need of care in the community [meso]	Services implement processes to identify older people in the community/defined geographical area who are in need of health and/or social care.	P: 1·2	P: 1·2	P: 6·0	P: 42·9	P: 48·8	P: 79·2
			LIC: 0	LIC: 0	LIC: 0	LIC: 0	LIC: 100·0	LIC: 100·0
			MIC: 3·3	MIC: 3·3	MIC: 6·7	MIC: 40·0	MIC: 46·7	MIC: 88·5
			HIC: 0	HIC: 0	HIC: 6·1	HIC: 49·0	HIC: 44·9	HIC: 71·7
13*	Services undertake comprehensive assessments when older people enter health or social care services where a decline in intrinsic capacity** is suspected or observed [meso]	Services implement processes to undertake comprehensive assessments of older people’s health and social care needs where a decline in intrinsic capacity** (IC) is suspected or observed (e.g. through a brief IC assessment). Assessments should be informed by the older person’s preferences, goals and physical and social environment and supported by developing or adapting existing tools, processes and guidelines.	P: 1·2	P: 2·4	P: 6·0	P: 32·1	P: 58·3	P: 93·4^#^
			LIC: 0	LIC: 0	LIC: 0	LIC: 0	LIC: 100·0	LIC: 100·0
			MIC: 3·3	MIC: 3·3	MIC: 6·7	MIC: 26·7	MIC: 60·0	MIC: 92·3
			HIC: 0	HIC: 2·0	HIC: 6·1	HIC: 38·8	HIC: 53·1	HIC: 93·3
14*	Services support appropriately trained health and social care workers to develop personalised care plans for older persons that are feasible, practical and target functional ability^ [meso]	Services support the development of single, personalised care plans for older persons based on a comprehensive assessment of their health (e.g. disease management) and social care needs as well as their goals and preferences. Where appropriate, care plans should also incorporate advance care planning and be revised as a person’s health/social circumstances change over time. Services provide health and social care professionals with appropriated tools, training (knowledge and skills-based competencies) and support systems (supervision, referral) to ensure that safe and quality care plans are created.	P: 1·2	P: 0	P: 6·0	P: 40·5	P: 52·4	P: 89·7^#^
			LIC: 0	LIC: 0	LIC: 0	LIC: 0	LIC: 100·0	LIC: 100·0
			MIC: 3·3	MIC: 0	MIC: 3·3	MIC: 46·7	MIC: 46·7	MIC: 89·3
			HIC: 0	HIC: 0	HIC: 8·2	HIC: 40·8	HIC: 51·0	HIC: 88·9
15*	Services establish networks of health and social care providers to enable timely referral and service provision [meso]	Services establish networks of local health and social care service providers to facilitate appropriate and timely on-referral to address health and/or social care needs of older people and their carers.	P: 1·2	P: 0	P: 2·4	P: 40·5	P: 56·0	P: 88·9^#^
			LIC: 0	LIC: 0	LIC: 0	LIC: 0	LIC: 100·0	LIC: 100·0
			MIC: 3·3	MIC: 0	MIC: 0	MIC: 43·3	MIC: 53·3	MIC: 89·7
			HIC: 0	HIC: 0	HIC: 4·1	HIC: 42·9	HIC: 53·1	HIC: 87·2
IPCHS Domain 5: Creating an enabling environment								
16*	Systems develop capacity in the current and emerging workforce (paid and unpaid) to deliver integrated care [macro]	Support the development of knowledge and skills to undertake comprehensive assessments, develop personalised care plans and deliver services that target functional ability^ in the current and emerging (students, trainees, or new or extended scope roles) health and social care workforce.	P: 1·2	P: 0	P: 1·2	P: 36·9	P: 60·7	P: 93·9^#^
			LIC: 0	LIC: 0	LIC: 0	LIC: 0	LIC: 100·0	LIC: 100·0
			MIC: 3·3	MIC: 0	MIC: 0	MIC: 43·3	MIC: 53·3	MIC: 100·0
			HIC: 0	HIC: 0	HIC: 2·0	HIC: 36·7	HIC: 61·2	HIC: 89·2
17*	Systems establish equitable human resource management processes to support the paid and unpaid workforce [macro]	Systems implement human resource management systems and processes to effectively manage and support the paid and unpaid workforce in an equitable manner across services. Processes need to be locally specific and suitable to the local context.	P: 1·2	P: 2·4	P: 7·1	P: 56·0	P: 33·3	P: 73·3
			LIC: 0	LIC: 0	LIC: 0	LIC: 0	LIC: 100·0	LIC: 100·0
			MIC: 3·3	MIC: 0	MIC: 0	MIC: 70·0	MIC: 26·7	MIC: 79·3
			HIC: 0	HIC: 4·1	HIC: 12·2	HIC: 53·1	HIC: 30·6	HIC: 65·9
18*	Systems utilise digital strategies to support self-management by older people [macro]	Where locally acceptable and feasible, systems implement digital strategies to support self-management of health conditions by older people.	P: 1·2	P: 2·4	P: 11·9	P: 56·0	P: 28·6	P: 52·1
			LIC: 0	LIC: 0	LIC: 0	LIC: 40·0	LIC: 60·0	LIC: 80·0
			MIC: 3·3	MIC: 0	MIC: 6·7	MIC: 56·7	MIC: 33·3	MIC: 55·6
			HIC: 0	HIC: 4·1	HIC: 16·3	HIC: 57·1	HIC: 22·4	HIC: 46·2
19*	Systems structure financing models to support integrated health and social care for older people [macro]	Systems establish financing policies and mechanisms to support integration of health and social care for older people though: i) joint/pooled health and social sector funding, managed at the system level, and/or ii) incentives for effective care coordination at the service level.	P: 1·2	P: 0	P: 2·4	P: 29·8	P: 66·7	P: 90·1^#^
			LIC: 0	LIC: 0	LIC: 0	LIC: 0	LIC: 100·0	LIC: 100·0
			MIC: 3·3	MIC: 0	MIC: 0	MIC: 33·3	MIC: 63·3	MIC: 86·2
			HIC: 0	HIC: 0	HIC: 4·1	HIC: 30·6	HIC: 65·3	HIC: 91·5

P: pooled responses (n = 84); LIC: low-income country responses (n = 5); MIC: middle-income country responses (n = 30); HIC: high-income country responses (n = 49).

* retained action based on threshold of ≥80% pooled agree + strongly agree response

# essential action based on threshold of ≥80% pooled responses as essential

^ *Functional ability*: the health related attributes that enable people to be and do what they have reason to value. It is made up of the intrinsic capacity of the individual, the relevant environmental characteristics and the interactions between the individual and these characteristics.

** *Intrinsic capacity*: the composite of person’s total physical and mental reserves that they may draw on.

**Fig 2:**
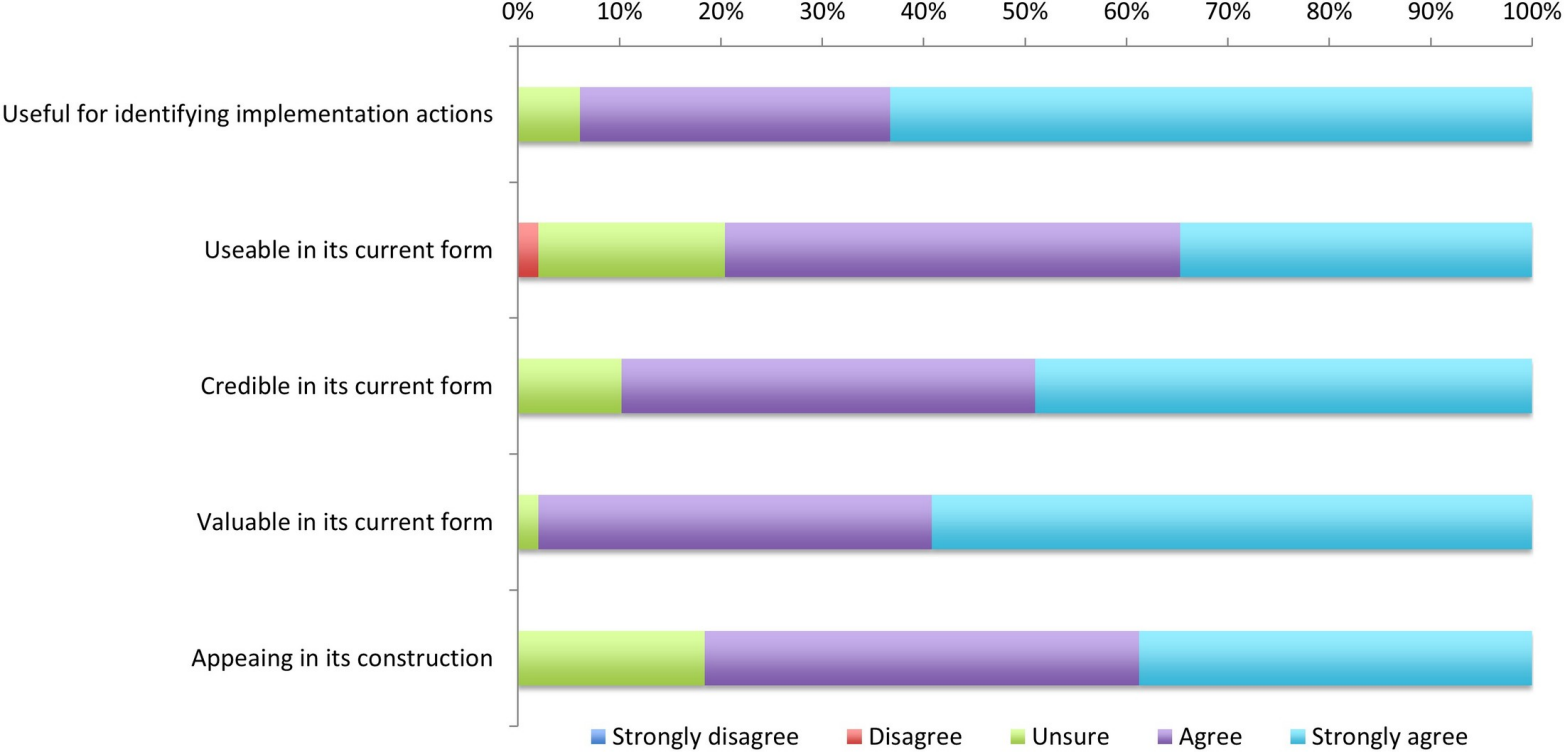
Outcomes across the domains of the Honeycomb User Experience Model,[25] plotted according to Likert scale categories.

The panel strongly supported the revised framework of actions with ratings of agree or strongly agree across the five dimensions of the User Experience model of 79·6–98·0% (Fig 2).

## Discussion

While previous literature has synthesised evidence for the components of integrated care approaches for older people and barriers and enablers to their implementation [5, 6], to our knowledge this is the first study to examine the specific actions required to implement integrated care at community level and identify actions needed in systems (macro-level) and services (meso-level) globally. WHO has already undertaken research at the micro level [20]. These data are essential for WHO to take evidence-informed actions in supporting implementation of the ICOPE approach in Member States. In particular, the actions will contribute to improving quality of care for older people, which is critical to achieving universal health coverage [27]. The final set of 19 actions (10 macro-level and 9 meso-level focussed) was strongly supported by the Delphi panel and the construction of the framework deemed highly useful, usable, credible, valuable and appealing according to the User Experience model. The specific actions guide policy makers and service managers to provide integrated care for older people through harnessing community involvement; building capacity in the paid and unpaid workforce; establishing appropriate governance, policy and financing models; prioritising service delivery in community settings; and levering health information and digital systems.

The Delphi panel was representative of a broad cross-section of experienced stakeholders across disciplines, care settings and geographies and was comparable between rounds 1 and 2. However, the panel was over-represented by research/academic participants and stakeholders from high-income settings in Asia and the European Union. Although this may introduce some sampling bias and influence the uptake and transferability of the final framework, we observed infrequent differences in the ranking of actions between high-, middle-, and low-income settings. It may be that with greater sampling of participants from low and middle-income settings, the frequency distributions may shift somewhat and this represents an important area for further research.

At the conclusion of round 1, three elements were flagged as uncertain, and none were excluded. This suggests that the initial, empirically derived set of 31 elements for ICOPE largely reflected contemporary approaches to integrated care for older people and the state of the evidence for interventions and implementation priorities. The uncertain elements (provider report cards, traditional and complementary medicines, new work cadres) were perhaps the most controversial of the initial set of 31 elements and insufficiently described. The additional elements identified by panellists from high-income settings as uncertain (case finding and performance management practices) may have also reflected a perception of overly burdensome system reform requirements for implementation of the ICOPE approach in systems that are already highly structured with relatively less flexibility than those of low- and middle-income settings. Notably, evidence synthesis suggests that macro-level contextual factors, such as cultural inertia and system instability; as well as specific provider-level factors in engagement, understanding and communication may stymie implementation efforts [6]. The opportunity to debate these uncertainties at the global consultation meeting facilitated a shared understanding of meaning of the elements and how they could be considered in implementing the ICOPE approach.

Round 2 outcomes confirmed consensus around a revised framework of 19 actions (re-framed from elements), with no actions eliminated, and support for the construction of the framework organised by *WHO Framework on integrated people-centred health services*. Although meeting the threshold for inclusion, the use of digital technologies to support self-management of health in older people (action #18) was ranked relatively the least important for implementing integrated care for older people, driven largely by responses from panellists in high-income settings. Conversely, panellists from middle- and low-income agreed more strongly that such digital technologies were needed to implement integrated care for older people. This geographic variance may indicate an increased awareness of the potential for remote monitoring, decision aids and communication offered by smart and mobile technologies [28, 29], on a background of widespread use of mobile phones in low- and middle-income settings offering opportunities at scale through mHealth technologies [30]. While not considered essential for implementation of the ICOPE approach, digital technologies are increasingly likely to support ageing in place and enable access to health services for older people, particularly as design moves towards a more person-centred focus [31].

Five actions were considered non-essential for implementation of the ICOPE approach. This outcome was largely influenced by respondents from high-income settings. Three actions related to information and communication technologies (ICT) and likely reflect concerns about capacity of health systems, particularly those in low- and middle-income settings, to implement and finance ICT systems with appropriate interoperability and security features, consistent with aligned evidence [32]. Respondents from low- and middle-income settings considered case finding processes essential, which may reflect greater care and access disparities for older people in these settings compared with high-income settings. High- and middle-income respondents did not consider human resources processes essential, perhaps due to the existence of such processes already in these settings. Nonetheless, the *WHO Global strategy on human resources for health* identifies human resources processes as a key component of workforce capacity building to meeting the Sustainable Development Goals [33].

While our findings relate to specific implementation actions for the WHO ICOPE approach, the domains align with evidence from prior reviews on components of integrated care for older adults and foci for implementation [5, 6], suggesting construct validity. Unlike other approaches supporting implementation of integrated care approaches, this framework particularly highlights the central role of unpaid carers, such as family members [34], as a critical component of the workforce, particularly in long-term care systems [35], and the need to appropriately support this workforce. Our data similarly reflect the view that older people and their families should be active partners in care planning and delivery across the care continuum to facilitate health system strengthening and its resilience [34]. Our research has a number of strengths. First, these data will inform WHO actions to support implementation of the ICOPE approach in Member States, reflecting a pre-planned, direct translation pathway to implementation tools that target meso and macro-level processes–levels that are largely underrepresented in implementation research [5, 6]. While evidence for components of integrated care interventions for older adults is evolving [5, 6], major gaps exist in the sustainable implementation of effective models into systems and services, likely due to the complexities of implementation [17, 18]. Second, we initiated the Delphi process with an empirically derived, evidence-informed framework of elements, rather than seeking expert opinion. Thus, we relied on experts to judge the feasibility and suitably of the elements, rather than the evidence underpinning them–creating an implementation rather than efficacy focus. Third, inclusion of a global consultation meeting between rounds 1 and 2 provided the opportunity to interpret and contextualise the findings from round 1, and develop a more user-centred and intuitive framework of actions in the subsequent round. The data presented should be interpreted in the context of some limitations. Sampling bias may represent an important limitation in this study. First there is a sampling bias towards panellists in high-income settings with oversampling of academic participants. Nonetheless, we achieved good representation and engagement from Member States, which will ultimately assist in supporting implementation efforts. In this context, it will be important to evaluate acceptability of any implementation tools developed by WHO by the anticipated end users of the research outcomes. It is important that future research and implementation efforts also specifically involve stakeholders from low and middle-income settings to ensure transferability of findings to these settings and to overcome limited research involving these settings [36]. Second, the sampling method did not include older people (consumers) as panel members. While we recognise the importance of appropriately including consumers in research, from inception through to implementation, the focus of the study was at the service and system levels with anticipated primary users of the research product/outcome being service and system-level managers/decision makers, thus not directly relevant to consumers. While existing research has identified consumers’ views on integrated care approaches [37, 38], it will be critical to involve consumers at all stages of implementation, as identified in final consensus framework of actions.

## Supporting information

S1 File Additional 15 elements proposed in Round 1. (DOCX)

S1 Table Summary of Round 1 outcomes. (DOCX)

## References

[1] World Health Organisation. World Report on Ageing and Health Geneva: WHO, 2015.

[2] Afshar S, Roderick PJ, Kowal P, Dimitrov BD, Hill AG. Multimorbidity and the inequalities of global ageing: a cross-sectional study of 28 countries using the World Health Surveys. BMC Public Health. 2015;15:776 10.1186/s12889-015-2008-7 26268536 PMC4534141

[3] World Health Organisation. Global strategy and action plan on ageing and health Geneva: World Health Organization, 2016.

[4] World Health Organization. Framework on integrated, people-centred health services Geneva: World Health Organization, 2016 Contract No.: A69/39.

[5] Briggs AM, Valentijn PP, Thiyagarajan JA, Araujo de Carvalho I. Elements of integrated care approaches for older people: a review of reviews. BMJ Open. 2018;8(4):e021194 10.1136/bmjopen-2017-021194 29627819 PMC5892746

[6] Threapleton DE, Chung RY, Wong SYS, Wong E, Chau P, Woo J, et al Integrated care for older populations and its implementation facilitators and barriers: A rapid scoping review. Int J Quality Health Care. 2017;29(3):327–34. 10.1093/intqhc/mzx041 28430963

[7] Araujo de Carvalho I, Epping-Jordan J, Pot AM, Kelley E, Toro N, Thiyagarajan JA, et al Organizing integrated health-care services to meet older people’s needs. Bull World Health Organ. 2017;95(11):756–63. 10.2471/BLT.16.187617 29147056 PMC5677611

[8] Beswick AD, Rees K, Dieppe P, Ayis S, Gooberman-Hill R, Horwood J, et al Complex interventions to improve physical function and maintain independent living in elderly people: a systematic review and meta-analysis. Lancet. 2008;371(9614):725–35. 10.1016/S0140-6736(08)60342-6 18313501 PMC2262920

[9] Bouman A, van Rossum E, Nelemans P, Kempen G, Knipschild P. Effects of intensive home visiting programs for older people with poor health status: A systematic review. BMC Health Serv Res. 2008;8 10.1186/1472-6963-8-74 18387184 PMC2364620

[10] Huss A, Stuck AE, Rubenstein LZ, Egger M, Clough-Gorr KM. Multidimensional preventive home visit programs for community-dwelling older adults: A systematic review and meta-analysis of randomized controlled trials. J Gerontol Ser A-Biol Sci Med Sci. 2008;63(3):298–307. 10.1093/gerona/63.3.298 18375879

[11] Stuck AE, Egger M, Hammer A, Minder CE, Beck JC. Home visits to prevent nursing home admission and functional decline in elderly people—Systematic review and meta-regression analysis. JAMA-J Am Med Assoc. 2002;287(8):1022–8. 10.1001/jama.287.8.1022 11866651

[12] Tappenden P, Campbell F, Rawdin A, Wong R, Kalita N. The clinical effectiveness and cost-effectiveness of home-based, nurse-led health promotion for older people: a systematic review. Health Techno Assess. 2012;16(20):1–72. 10.3310/hta16200 22490205 PMC4781606

[13] van Haastregt JCM, Diederiks JPM, van Rossum E, de Witte LP, Crebolder H. Effects of preventive home visits to elderly people living in the community: systematic review. Br Med J. 2000;320(7237):754–8. 10.1136/bmj.320.7237.754 10720360 PMC27318

[14] You EC, Dunt D, Doyle C, Hsueh A. Effects of case management in community aged care on client and carer outcomes: a systematic review of randomized trials and comparative observational studies. BMC Health Serv Res. 2012;12 10.1186/1472-6963-12-395 23151143 PMC3508812

[15] Valentijn PP, Boesveld IC, van der Klauw DM, Ruwaard D, Struijs JN, Molema JJ, et al Towards a taxonomy for integrated care: a mixed-methods study. Int J Integr Care. 2015;15:e003 25759607 10.5334/ijic.1513 PMC4353214

[16] Valentijn PP, Schepman SM, Opheij W, Bruijnzeels MA. Understanding integrated care: a comprehensive conceptual framework based on the integrative functions of primary care. Int J Integr Care. 2013;13:e010 23687482 10.5334/ijic.886 PMC3653278

[17] Angus L, Valentijn PP. From micro to macro: assessing implementation of integrated care in Australia. Aust J Prim Health. 2018;24(1):59–65. 10.1071/PY17024 29132497

[18] Kirst M, Im J, Burns T, Baker GR, Goldhar J, O'Campo P, et al What works in implementation of integrated care programs for older adults with complex needs? A realist review. Int J Quality Health Care. 2017;29(5):612–24. 10.1093/intqhc/mzx095 28992156 PMC5890872

[19] Briggs AM, Chan M, Slater H. Models of Care for musculoskeletal health: Moving towards meaningful implementation and evaluation across conditions and care settings. Best Pract Res Clin Rheumatol. 2016;30(3):359–74. 10.1016/j.berh.2016.09.009 27886937

[20] World Health Organization. Guidelines on community-level interventions to manage declines in intrinsic capacity Geneva: WHO, 2017. 29608259

[21] World Health Organization. Global consultation on integrated care for older people (ICOPE)–the path to universal health coverage: report of consultation meeting 23–25 October 2017 in Berlin, Germany Geneva: WHO, 2018.

[22] Fitch K, Bernstein SJ, Aguilar MA, Burnard B, Ramon LaCalle J, van het Loo M, et al The RAND/UCLA Appropriateness Method User’s Manual Santa Monica, CA: RAND; 2001.

[23] Hsieh HF, Shannon SE. Three approaches to qualitative content analysis. Qual Health Res. 2005;15(9):1277–88. 10.1177/1049732305276687 16204405

[24] Giannarou L, Zervas E. Using Delphi technique to build consensus in practice. Int J Business Science Applied Management. 2014;9(2):65–82.

[25] Moreville P. User Experience Design Michigan: Semantic Studios; 2014 [cited 2016 5 January]. Available from: http://semanticstudios.com/user_experience_design/.

[26] Briggs AM, Jordan JE, Jennings M, Speerin R, Bragge P, Chua J, et al Supporting the evaluation and implementation of musculoskeletal Models of Care: A globally informed framework for judging readiness and success. Arthritis Care Res. 2017;69(4):567–77. 10.1002/acr.22948 27273891

[27] World Health Organization., Organisation for Economic Co-operation and Development., The World Bank. Delivering quality health services: a global imperative for universal health coverage Geneva: WHO, OECD and The World Bank, 2018.

[28] Liu LL, Stroulia E, Nikolaidis I, Miguel-Cruz A, Rincon AR. Smart homes and home health monitoring technologies for older adults: A systematic review. Int J Med Inform. 2016;91:44–59. 10.1016/j.ijmedinf.2016.04.007 27185508

[29] Free C, Phillips G, Watson L, Galli L, Felix L, Edwards P, et al The effectiveness of mobile-health technologies to improve health care service delivery processes: A systematic review and meta-analysis. PLOS Medicine. 2013;10(1):e1001363 10.1371/journal.pmed.1001363 23458994 PMC3566926

[30] Davey S, Davey A. Mobile-health technology: Can it strengthen and improve public health systems of other developing countries as per Indian strategies? A systematic review of the literature. Int J Med Public Health. 2014;4(1):40–5.

[31] Wildenbos GA, Peute L, Jaspers M. Aging barriers influencing mobile health usability for older adults: A literature based framework (MOLD-US). Int J Med Inform. 2018;114:66–75. 10.1016/j.ijmedinf.2018.03.012 29673606

[32] Slater H, Campbell JM, Stinson JN, Burley MM, Briggs AM. End user and implementer experiences of mHealth technologies for noncommunicable chronic disease management in young adults: Systematic review. J Med Internet Res. 2017;19(12):e406 10.2196/jmir.8888 29233804 PMC5743925

[33] World Health Organization. Global strategy on human resources for health: Workforce 2030 Geneva: WHO, 2016.

[34] O'Hara JK, Aase K, Waring J. Scaffolding our systems? Patients and families 'reaching in' as a source of healthcare resilience. BMJ Qual Saf. 2018 10.1136/bmjqs-2018-008216 29764929

[35] Pot AM, Briggs AM, Beard JR. The sustainable development agenda needs to include long-term care. J Am Med Dir Assoc. 2018;19(9):725–7. 10.1016/j.jamda.2018.04.009 30149838

[36] Lloyd-Sherlock P. Beyond neglect: Long-term care research in low and middle income countries. Int J Gerontol. 2014;8(2):66–9. 10.1016/j.ijge.2013.05.005.

[37] D'Avanzo B, Shaw R, Riva S, Apostolo J, Bobrowicz-Campos E, Kurpas D, et al Stakeholders' views and experiences of care and interventions for addressing frailty and pre-frailty: A meta-synthesis of qualitative evidence. PLoS ONE. 2017;12(7):e0180127 10.1371/journal.pone.0180127. 28723916 PMC5516973

[38] Spoorenberg SLW, Wynia K, Fokkens AS, Slotman K, Kremer HPH, Reijneveld SA. Experiences of community-living older adults receiving integrated care based on the Chronic Care Model: A qualitative study. Plos One. 2015;10(10). 10.1371/journal.pone.0137803 26489096 PMC4619446

