# Supplementary material for: Actions required to implement integrated care for older people in the community using the World Health Organization's ICOPE approach: A global Delphi consensus study
Source: PLoS One. 2018 Oct 11;13(10):e0205533. doi: 10.1371/journal.pone.0205533 (PMC6181385; doi:10.1371/journal.pone.0205533)
Supplement: S1 File — (DOCX) [file pone.0205533.s001.docx]

**S1 File. Additional 15 elements proposed in Round 1**

Panellists cited the following elements as important considerations, based on free-text responses:

1. Telemedicine support
2. Home-hospital services to avoid emergency admissions
3. Integrated medicine prescription systems
4. Engagement of older people in good health in the care of their peers
5. Specialised services in hospitals, such as geriatric medicine departments
6. Boundary-spanning reimbursement models, where different actors are jointly responsible for the results
7. Special care administrative and legal frameworks to protect older persons from abuse
8. Technologies to support self-monitoring of health
9. Formal and sensitive mechanisms for direct feedback from service providers
10. Availability of evidence-based clinical guidelines to avoid delivery of interventions that are not evidence-based
11. A protocol to guide engagement with the private sector
12. Establishment of supervisor position(s) and timely support processes for frontline health workers in diagnosis and referral planning
13. Financial benefits for provision of appropriate care
14. Implementation of a dedicated long-term care system
15. Age-friendly infrastructure
