# Supplementary material for: Actions required to implement integrated care for older people in the community using the World Health Organization's ICOPE approach: A global Delphi consensus study
Source: PLoS One. 2018 Oct 11;13(10):e0205533. doi: 10.1371/journal.pone.0205533 (PMC6181385; doi:10.1371/journal.pone.0205533)
Supplement: S1 Table — (DOCX) [file pone.0205533.s002.docx]

**S1 Table. Summary of Round 1 outcomes**

| **Element** | | **Total panel (n=80)** | | | **High-income (n=56)** | | | **Middle-income (n=16)** | | | **Low-income (n=8)** | | |
| --- | --- | --- | --- | --- | --- | --- | --- | --- | --- | --- | --- | --- | --- |
|  |  | **Median (IQR)** | **% agree in median band^** | **Consensus** | **Median (IQR)** | **% agree in median band^** | **Consensus** | **Median (IQR)** | **% agree in median band^** | **Consensus** | **Median (IQR)** | **% agree in median band^** | **Consensus** |
| **1** | Individualised interdisciplinary care plans are available for older people | 8 (1) | 93.7 | Important | 8 (1) | 91.1 | Important | 8.5 (1) | 100.0 | Important | 9 (2) | 100.0 | Important |
| **2** | Active case finding is undertaken | 8 (3) | 72.5 | Important | 7 (3) | 66.1 | Uncertain | 8 (1) | 88.2 | Important | 9 (2) | 85.7 | Important |
| **3** | Older people enter a common assessment and care pathway regardless of the entry point into the health system | 7 (2) | 70.0 | Important | 7 (2) | 70.1 | Important | 7.5 (3) | 70.5 | Important | 7.5 (3) | 71.4 | Important |
| **4** | Community-based health workers are available | 8 (1.75) | 96.2 | Important | 8 (2) | 96.4 | Important | 9 (1) | 94.1 | Important | 8.5 (2) | 100.0 | Important |
| **5** | Care is delivered by interdisciplinary teams | 8 (1) | 90.0 | Important | 8 (1) | 87.5 | Important | 8 (1) | 94.1 | Important | 9 (2) | 100.0 | Important |
| **6** | Community-based care services are available | 8 (1) | 92.5 | Important | 8 (2) | 92.8 | Important | 8 (1) | 94.1 | Important | 9 (0) | 85.7 | Important |
| **7** | Civil society (NGOs) and patient groups are involved at the community level | 8 (2.75) | 75.0 | Important | 8 (3) | 71.4 | Important | 8 (1) | 88.2 | Important | 8.5 (3) | 71.4 | Important |
| **8** | Training and support for formal and informal carers | 8 (1) | 82.5 | Important | 8 (1) | 82.1 | Important | 8 (2) | 76.4 | Important | 8 (2) | 100.0 | Important |
| **9** | Respite care is available | 7 (2) | 70.0 | Important | 7 (3) | 70.0 | Important | 8 (4) | 71.0 | Important | 7 (2) | 71.4 | Important |
| **10** | Communities are engaged in shaping care systems | 8 (2) | 86.2 | Important | 8 (2) | 83.9 | Important | 9 (1) | 100.0 | Important | 9 (3) | 71.4 | Important |
| **11** | Patients are involved in care decisions and planning | 9 (1) | 90.0 | Important | 9 (1) | 89.3 | Important | 8 (1) | 94.1 | Important | 9 (1) | 87.7 | Important |
| **12** | Provider report cards are used | 7 (3) | 51.2 | Uncertain | 6 (2) | 39.3 | Uncertain | 8 (2) | 70.5 | Important | 8 (1) | 100.0 | Important |
| **13** | Patients have the opportunity to report their experiences | 8 (2) | 81.2 | Important | 8 (2) | 76.7 | Important | 8 (2) | 94.1 | Important | 8.5 (2) | 85.7 | Important |
| **14** | Patient-reported outcome measures (PROMS) are monitored | 8 (2) | 85.0 | Important | 8 (2) | 82.1 | Important | 8 (1) | 88.2 | Important | 8 (2) | 100 | Important |
| **15** | Community-based home care services are available | 8 (2) | 91.2 | Important | 8 (1) | 91.1 | Important | 8 (1) | 94.1 | Important | 9 (2) | 85.2 | Important |
| **16** | Inter-professional education is routinely available | 8 (2) | 81.2 | Important | 7.5 (3) | 73.2 | Important | 9 (1) | 100.0 | Important | 8 (1) | 100.0 | Important |
| **17** | Inter-professional governance frameworks are in place | 8 (2) | 82.5 | Important | 8 (2) | 78.5 | Important | 8 (2) | 88.2 | Important | 9 (1) | 100.0 | Important |
| **18** | Traditional and complementary medicine is integrated within health services | 6 (3) | 40.0 | Uncertain | 6 (3) | 32.1 | Uncertain | 7 (3) | 70.6 | Important | 6 (2) | 28.5 | Uncertain |
| **19** | Performance management practices are established for care providers | 7.5 (1) | 76.2 | Important | 7 (2) | 67.8 | Uncertain | 8 (2) | 94.1 | Important | 8 (2) | 100.0 | Important |
| **20** | Physical infrastructure to support community-based health and social care providers is available | 8 (2) | 86.2 | Important | 8 (1) | 80.3 | Important | 9 (1) | 100.0 | Important | 9 (0) | 100.0 | Important |
| **21** | New work cadres are developed | 7 (2) | 65.0 | Uncertain | 7 (2) | 62.5 | Uncertain | 8 (4) | 64.7 | Uncertain | 8 (2) | 85.7 | Important |
| **22** | Planning and delivery of health and social care are integrated | 8 (1) | 95.0 | Important | 8 (2) | 92.8 | Important | 9 (1) | 100.0 | Important | 9 (2) | 100.0 | Important |
| **23** | Human resource management is aligned across services | 8 (2) | 87.5 | Important | 7.5 (1) | 82.1 | Important | 8 (2) | 100.0 | Important | 8.5 (2) | 100.0 | Important |
| **24** | Electronic data sharing platforms are in place | 8 (2) | 83.7 | Important | 8 (2) | 89.3 | Important | 8 (2) | 82.3 | Important | 9 (1) | 85.7 | Important |
| **25** | Regular feedback of performance indicators is given to care providers | 8 (1) | 85.0 | Important | 8 (1) | 80.3 | Important | 8 (2) | 100.0 | Important | 9 (1) | 85.7 | Important |
| **26** | Joint funding mechanisms for health and social care are in place | 8 (2) | 80.0 | Important | 7 (3) | 75.0 | Important | 8 (2) | 88.2 | Important | 8.5 (1) | 100.0 | Important |
| **27** | The capacity of health and social care systems to deliver integrated care is regularly assessed | 8 (1.75) | 83.7 | Important | 8 (1) | 76.8 | Important | 8 (1) | 100.0 | Important | 8 (1) | 100.0 | Important |
| **28** | Assistive products (devices, technologies) are available | 8 (2) | 78.7 | Important | 8 (3) | 75.0 | Important | 8 (1) | 88.2 | Important | 8.5 (2) | 85.7 | Important |
| **29** | Networks of health providers are established to facilitate referral pathways, including pathways for rapid access t acute care | 8 (2) | 88.7 | Important | 8 (2) | 85.7 | Important | 9 (1) | 100.0 | Important | 8.5 (1) | 85.7 | Important |
| **30** | Incentives are in place for care co-ordination | 8 (2) | 81.2 | Important | 8 (2) | 82.1 | Important | 8 (3) | 76.4 | Important | 8 (2) | 85.7 | Important |
| **31** | A regulatory framework is in place | 8 (2) | 85.0 | Important | 7 (2) | 78.5 | Important | 8 (1) | 100.0 | Important | 9 (1) | 100.0 | Important |

NGOs: non-governmental organizations
^ median bands defined as per RAND UCLA criteria: 1-3; 4-6; 7-9
